# Single versus Dual-Operator Approaches for Percutaneous Coronary Interventions within Chronic Total Occlusion—An Analysis of 27,788 Patients

**DOI:** 10.3390/jcm12144684

**Published:** 2023-07-14

**Authors:** Rafał Januszek, Giuseppe De Luca, Wojciech Siłka, Leszek Bryniarski, Krzysztof Piotr Malinowski, Andrzej Surdacki, Wojciech Wańha, Stanisław Bartuś, Aleksandra Piotrowska, Krzysztof Bartuś, Kamil Pytlak, Zbigniew Siudak

**Affiliations:** 1 Department of Cardiology and Cardiovascular Interventions, University Hospital, 30-688 Kraków, Poland; rjanuszek@su.krakow.pl (R.J.); l_bryniarski@poczta.fm (L.B.); andrzej.surdacki@uj.edu.pl (A.S.); stanislaw.bartus@uj.edu.pl (S.B.); 2 Division of Cardiology, AOU Policlinico G. Martino, University of Messina, 98166 Messina, Italy; giuseppe.deluca@med.uniupo.it; 3 Division of Cardiology, IRCCS Hospital Galeazzi-Sant’Ambrogio, 20161 Milan, Italy; 4 Department of Bioinformatics and Telemedicine, Faculty of Medicine, Jagiellonian University Medical College, 31-008 Kraków, Poland; krzysztof.piotr.malinowski@gmail.com; 5 Institute of Cardiology, Jagiellonian University Medical College, 31-008 Kraków, Poland; 6 Department of Cardiology and Structural Heart Diseases, Medical University of Silesia, 40-635 Katowice, Poland; wojciech.wanha@gmail.com; 7 Faculty of Medicine and Health Sciences, Jan Kochanowski University, 25-369 Kielce, Poland; alemarpiotrowska@gmail.com (A.P.); pytlak.kam@gmail.com (K.P.); zbigniew.siudak@gmail.com (Z.S.); 8 Department of Cardiovascular Surgery and Transplantology, Jagiellonian University Medical College, John Paul II Hospital, 31-202 Kraków, Poland; krzysztofbartus@gmail.com

**Keywords:** chronic total occlusion, dual operator, single operator

## Abstract

(1) Background: Since the treatment of chronic total occlusion (CTO) with percutaneous coronary intervention (PCI) is associated with high procedural complexity, it has been suggested to use a multi-operator approach. This study was aimed at evaluating the procedural outcomes of single (SO) versus dual-operator (DO) CTO-PCI approaches. (2) Methods: This retrospective analysis included data from the Polish Registry of Invasive Cardiology Procedures (ORPKI), collected between January 2014 and December 2020. To compare the DO and SO approaches, propensity score matching was introduced with equalized baseline features. (3) Results: The DO approach was applied in 3604 (13%) out of 27,788 CTO-PCI cases. Patients undergoing DO CTO-PCI experienced puncture-site bleeding less often than the SO group (0.1% vs. 0.3%, *p* = 0.03). No differences were found in the technical success rate (successful revascularization with thrombolysis in myocardial infarction flow grade 2/3) of the SO (72.4%) versus the DO approach (71.2%). Moreover, the presence of either multi-vessel (MVD) or left main coronary artery disease (LMCA) (odds ratio (OR), 1.67 (95% confidence interval (CI), 1.20–2.32); *p* = 0.002), as well as lower annual and total operator volumes of PCI and CTO-PCI, could be noted as factors linked with the DO approach. (4) Conclusions: Due to the retrospective character, the findings of this study have to be considered only as hypothesis-generating. DO CTO-PCI was infrequent and was performed on patients who were more likely to have LMCA lesions or MVD. Operators collaboratively performing CTO-PCIs were more likely to have less experience. Puncture-site bleeding occurred less often in the dual-operator group; however, second-operator involvement had no impact on the technical success of the intervention.

## 1. Introduction

At present, percutaneous coronary interventions (PCIs) are becoming more challenging. Such a situation is primarily due to the patient’s advanced age, complex anatomic lesions, and greater burden of concomitant diseases [1,2,3,4]. In turn, in selected groups of patients, this accounts for the higher complexity of the intervention, greater amount of contrast, and radiation dose used during the procedure. This further reflects longer procedural time, which is followed by an increased risk of adverse outcomes [3,4]. This is extremely relevant in PCIs performed within chronic total occlusions (CTOs), which are linked with more challenging procedures. Compared to regular PCIs, they are also connected with a higher risk of periprocedural complications, such as coronary artery perforation and loss of collateral circulation [5,6,7,8,9,10]. Higher complexity can also be caused by the poor condition of patients. Seeing as CTO may be present in many patients with acute coronary syndromes, patient prognosis becomes further exacerbated [11]. To address this issue, it has been recommended to perform high-risk PCIs. These include CTO-PCIs with a multiple-operator approach. This is performed in order to improve procedural success and safety [9,12,13,14]. The involvement of a second operator, depending on his/her experience, may provide support for the leading operator. This allows shared intra-procedural decision-making while simultaneously yielding educational benefits for the assistant operator [9]. However, in recently published studies on both high-risk PCIs and CTO-PCIs, no improvement has been reported in terms of procedural outcomes or major adverse cardiac event (MACE) rates among patients treated via the multiple-operator approach [4,15]. Nonetheless, since the latter remains poorly studied, we aimed to identify factors associated with single- (SO) and dual-operator (DO) approaches for CTO-PCIs and their impact on procedural outcomes.

## 2. Materials and Methods

### 2.1. Materials

This retrospective analysis is based on data from the national registry of percutaneous coronary interventions (ORPKIs), collected between 2014 and 2021. The registry is maintained in cooperation with the Association of Cardiovascular Interventions (AISNs) of the Polish Cardiac Society. It covers almost all catheter laboratories (CathLabs) performing PCIs in Poland and has been characterized in previously published papers [8]. We extracted 27,788 patients who had undergone either SO (*n* = 24,184) or DO (*n* = 3604) CTO-PCI between January 2014 and December 2020. The exact percentage share of PCI CTO in the entire population of patients undergoing PCI procedures in subsequent years has been presented in previous publications [6,8]. Due to the retrospective nature and anonymization of the collected data in the registry, obtaining the consent of the Bioethics Committee was waived.

### 2.2. Definitions

CTO was defined by coronary angiography as a coronary occlusion without antegrade filling of the distal vessel other than via collaterals assessed by thrombolysis in myocardial infarction (TIMI) at grade 0. The duration of the occlusion had to be more than 3 months, as estimated from the onset of clinical events, including myocardial infarction (MI), sudden onset or worsening of chest symptoms, and angiography. This further had to be confirmed by an experienced operator. According to the CTO-ARC consensus, technical success of the performed CTO-PCI was defined as restoration of coronary artery patency assessed by TIMI scale grade 2/3 with <30% residual stenosis of the target CTO lesion [16].

### 2.3. Study Endpoints

The primary endpoint of this study was the technical success of the performed CTO-PCI. Secondary endpoints included periprocedural complications, i.e., coronary artery perforation, puncture-site bleeding, MI, cardiac arrest, no-reflow phenomenon, or death. With regard to the aforementioned endpoints, the procedural outcomes were compared in the SO vs. DO study groups.

### 2.4. Statistical Analysis

Continuous variables are presented as mean + standard deviation or median (interquartile range), depending on normality assessed using the Shapiro–Wilk test. Categorical variables are presented as numeric values (percentages). Continuous variables were compared using Student’s and Welch’s *t*-tests, or Wilcoxon tests where necessary. Categorical variables were evaluated via the χ^2^ test or Fisher’s exact test when appropriate, with corresponding post hoc tests.

All factors, including patient medical history and procedure indices, were considered potential factors associated with the dual-operator approach for CTO-PCI. The appropriate univariable analyses were performed. Moreover, a multivariable regression model was constructed with outcomes as dependent variables (any periprocedural complication, no-reflow phenomenon, cardiac arrest, coronary artery perforation, death, allergic reaction, and MI), as well as the following independent variables: gender, diabetes, previous stroke, previous MI, previous PCI, previous coronary artery bypass graft (CABG), smoking status, psoriasis, hypertension, kidney disease, chronic obstructive pulmonary disease (COPD), Killip class IV, age, weight, contrast used in PCI, radiation dose used in PCI, annual site volume, annual operator volume, annual operator CTO volume, and DO approach. The association between the DO vs. SO approaches and outcome was adjusted for all independent variables listed above. The variance inflation factor was used to assess the multicollinearity in the multivariable model and select factors with greater impact in case of VIF value >10. To analyze the differences between the DO and the SO groups, propensity score matching was performed with the following variables selected for matching: age, contrast used in PCI, radiation dose used in PCI, weight, annual operator CTO volume, annual site volume, access site during PCI, gender, diabetes, previous stroke, previous MI, previous PCI, previous CABG, smoking status, hypertension, kidney disease, COPD, PCI within bifurcation, implanted stent, acetylsalicylic acid use, unfractionated heparin (UFH) use, low molecular weight heparin (LMWH) use, intravascular ultrasound during PCI, fractional flow reserve assessment during PCI, and optical coherent tomography during PCI. Due to the matching and partial lack of data, the propensity score-matched population consisted of 15,950 patients. A *p*-value lower than 0.05 was considered significant. Statistical analyses were performed using the following packages: R version 3.5.3, ‘lme4′ version 1.1-21, and ‘tidyverse’ version 1.2.1.

## 3. Results

This analysis included 27,788 CTO-PCIs, out of which 3604 (13%) were performed by 2 operators and 24,184 (87%) by 1 operator.

### 3.1. Clinical Characteristics at Baseline

The clinical characteristics at the baseline are shown in Table 1 and Table 2. The propensity score matched the yielded pairs of patients undergoing either DO CTO-PCI or SO CTO-PCI at a 1 to 4 ratio, respectively. The baseline characteristics were balanced with no significant differences.

### 3.2. Procedural Indices, Pharmacotherapy, and Procedural Outcomes

Most importantly, the technical success rate was similar in the DO and SO groups (71.2% and 72.4%, respectively, Table 3). Furthermore, CTO-PCIs in the DO cases were characterized by significantly higher usage of contrast volume and radiation dose (*p* < 0.0001) (Table 3). Furthermore, in these cases, access site crossover occurred more often (*p* = 0.02) and overall, femoral access was used more frequently (*p* < 0.0001). In comparison to the DO procedures, SO CTO-PCIs were performed at sites of greater annual and total volumes and by more experienced operators. Their values were expressed as the total and annual volume of the performed PCIs, including CTO cases (Table 3). However, as shown in Table 4, in the propensity score-matched population, only differences in contrast, radiation dose, and UFH reached statistical significance.

### 3.3. Procedure-Related Complications

Although no differences were found among the unmatched population, after propensity score matching, it was revealed that more patients experienced puncture-site bleeding in the SO group (Table 5 and Table 6).

**Figure 1:**
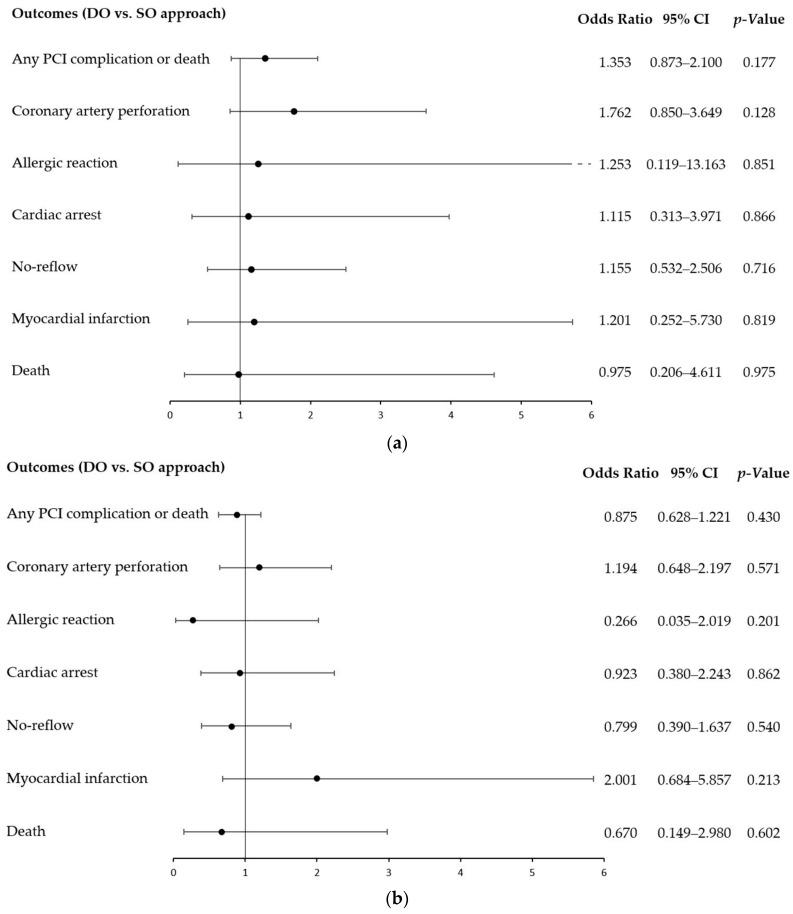
The dual-operator approach and procedure-related complications: multivariable analysis by outcomes for the unmatched (**a**) and propensity score-matched population (**b**). The association between the DO vs. SO approaches and outcomes were adjusted for gender, diabetes, previous stroke, previous myocardial infarction, previous PCI, previous coronary artery bypass graft, smoking status, psoriasis, hypertension, kidney disease, chronic obstructive pulmonary disease, Killip class IV, age, weight, contrast used in PCI, radiation dose used in PCI, annual site volume, annual operator volume, and annual operator CTO volume. CI, confidence interval; DO, dual operator; PCI, percutaneous coronary intervention; SO, single operator.

Furthermore, multivariable analysis revealed no statistically significant differences in the associations between DO vs. SO approach and procedural outcomes in both, unmatched and propensity score-matched population. (Figure 1).

### 3.4. Factors Associated with Dual-Operator CTO-PCI

**Figure 2:**
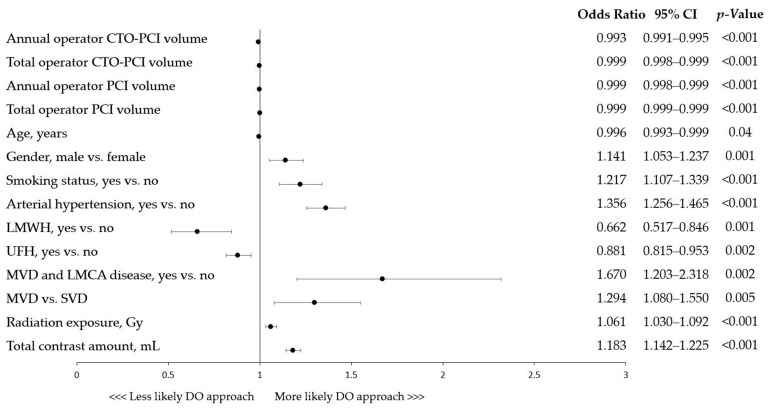
Factors associated with dual-operator CTO-PCI: univariable analyses. CI, confidence interval; CTO, chronic total occlusion; DO, dual operator; LMCA, left main coronary artery; LMWH, low molecular weight heparin; MVD, multi-vessel disease; PCI, percutaneous coronary intervention; SVD, single-vessel disease; UFH, unfractionated heparin.

The univariable analyses revealed that older age was not linked with CTO-PCIs performed by two operators. However, males (odds ratio (OR), 1.14 (95% confidence interval (CI), 1.05–1.24); *p* = 0.001) and current smokers (OR, 1.22 (95% CI, 1.11–1.34); *p* < 0.001), had higher odds of being treated by two CTO-PCI operators. This was also true for patients burdened with arterial hypertension (OR, 1.36 (95% CI, 1.26–1.47); *p* < 0.001) (Figure 2). Multi-vessel disease (MVD), in comparison to single-vessel disease (SVD) (OR, 1.30 (95% CI, 1.08–1.55); *p* = 0.005), and having either MVD or left main coronary artery (LMCA) involvement compared to the absence of such angiographic findings (OR, 1.67 (95% CI, 1.20–2.32); *p* = 0.002), were also associated with the DO approach. Furthermore, greater contrast amount and radiation dose used during the procedure itself were linked with DO CTO-PCI. Considering operators’ experience, those with lower annual and total volumes of both CTO-PCIs and overall PCIs had higher odds of performing CTO-PCI with a second operator. In contrast, patients administrated with UFH and LMWH had lower odds of being treated by two operators (Figure 2).

## 4. Discussion

To summarize the results of this study, the frequency of DO CTO-PCIs was 13%. This approach, however, was not associated with the improvement of procedural outcomes, i.e., technical success and periprocedural complication occurrence. Thirdly, CTO-PCIs in the DO group may have been more complex. This was due to the higher contrast and radiation doses used during the procedure. Such a situation concerned patients with greater MVD lesion rates as well as more frequent involvement of LMCA. Lastly, DO interventions were performed by operators who were more likely to be less experienced.

The revascularization of CTO lesions has been recognized as a great challenge for the operator. This is because it is linked with complex anatomic lesions demanding scrupulous vessel preparation, followed by longer procedural time, higher radiation dose, and contrast amount used during the intervention [3,6,17,18]. Apart from this, high procedural difficulty is attributed to a higher rate of additional device exploitation (e.g., intravascular imaging, rotablation, intravascular lithotripsy) compared to regular PCI. Moreover, challenging maneuvers, involving antegrade or retrograde dissection and re-entry approaches, may strain the mental and skillset capacity of the interventionalist [17,19]. Furthermore, the increasing clinical severity of patients eligible for PCI and its low predictability but high risk makes it more difficult to single-handedly manage these cases at the Cathlab [20]. Despite the aforementioned challenges, due to novel interventional therapies, the international procedural success of CTO-PCI has been steadily increasing over time, from less than 75% in the past to a current total of 90% at the leading highly experienced centers [9,17,20,21,22]. However, this outcome not only remains significantly worse in comparison to regular PCI, but also exhibits great variability with regard to the site where CTO treatment is attempted. In fact, in other studies based on inexperienced institutions, it has been reported that achieved procedural success is unsatisfactory, far below 70% [10,17]. Thus, in an effort to improve the modest outcomes of such revascularizations, a renewed interest has arisen in establishing international guidelines for CTO-PCIs and adopting widely convenient training programs. This involves, among other aspects, a multi-operator approach to CTO cases as well as other high-risk PCIs [9,13,14]. Indeed, the growing usage of the DO approach has been observed in the past years. It primarily concerns institutions with high annual PCI volumes, where LMCA disease, calcific stenosis, and CTO account for a great proportion of attempted cases. Also, imaging techniques, rotablation, and mechanical support devices are vividly exploited at these centers [4].

In our study, DO CTO-PCI was performed on patients with greater disease severity, as they were more likely to have LMCA lesions or MVD. Also, DO intervention was linked with a higher amount of contrast as well as radiation dose used during the procedure. Such findings are comparable to other reports on high-risk PCIs. They can further be explained by the preselection of complex cases during collaborative preplanning of the procedure, since in other studies, an association has been reported between contrast used during CTO-PCI and a greater rate of periprocedural complications and utilization of advanced devices and complex lesions, such as restenosis, i.e., all factors being commonly attributed to higher procedural difficulty [4,6]. In a different study on CTO-PCIs, Karacsonyi et al. showed that multi-operator procedures were also characterized by greater procedural and fluoroscopy time, as well as higher air kerma radiation dose and contrast volume, compared to the SO group [15]. However, in contrast to our results, patients treated by multi-operator CTO-PCI had lower lesion complexity. This was confirmed by a lower Japan-CTO (J-CTO) score (2.28 ± 1.20 vs. 2.38 ± 1.29; *p* = 0.005; respectively) and Prospective Global Registry for the Study of Chronic Total Occlusion Intervention (PROGRESS-CTO) (0.97 ± 0.93 vs. 1.13 ± 1.01; *p* < 0.001; respectively) [15]. It should also be noted that the increased amount of contrast and radiation dose may be related not only to the complexity of the lesions undergoing PCI but also the lack of experience of the operators performing such a procedure.

In our study, operator annual and total volume of all performed PCIs and CTO cases were identified as factors linked with the SO approach in the multivariable analysis. These findings seem to be similar to those obtained in other studies. In them, it has been reported that operators performing multi-operator high-risk procedures, including CTO cases, were less often highly experienced (>60 CTO cases per year), had fewer years of experience in PCIs, and had lower annual PCI volumes, including high-risk interventions [4,15]. One could hypothesize that the central tendency of operators’ experience in DO procedures could have been lowered by the participation of inexperienced juniors. However, the aforementioned studies differentiated the experience of leading interventionalists performing DO CTO-PCIs and in them, their superiority over SO operators was still not noted [4,15].

Most importantly, no improvement was demonstrated in the technical success or periprocedural complication rates in the DO group compared to SO. Coherently, in the study on high-risk PCIs, Kovach et al. reported similar outcomes between such two groups regarding the MACE rate (32% vs. 30%, *p* = 0.44) and its components at the 12-month follow-up. Acute kidney injury incidence, hospital length, and 30-day re-admission were also considered [4]. The paucity of anticipated outcome improvement may have been due to the biased group selection. The potential benefits of a second operator in terms of procedural success could have been confounded by the too-broad group selection, since they may appear only in the most complex cases, such as among patients with J-CTO scores >3. However, in the study based on the PROGRESS-CTO registry, no differences were noted in any J-CTO groups regarding the MACE rate (2.2% vs. 2.4%; *p* = 0.6), technical (86% vs. 86%; *p* = 0.9), or procedural success (84% vs. 85%; *p* = 0.6). Moreover, patients with PROGRESS CTO scores of 3 and 4, treated by multiple operators, had a significantly lower technical success rate compared to the SO group [15].

In part, the lack of procedural improvement could have also been driven by the fact that in some cases, PCIs performed by two operators were already linked with worse outcomes. That would appear as a consequence of the scenario in which an additional operator was called to aid the ongoing procedure following the occurrence of a sudden complication; thus, falsely accounting for a certain percentage of the DO group. Moreover, since the average technical success achieved in this registry is relatively low, it may be that the DO approach is a result of cooperation between two, non-dedicated CTO operators. In such a case, the potential benefits of an additional operator were countered by the greater experience of interventionalists single-handedly performing CTO-PCIs at centers with higher volumes of CTO interventions. In multiple studies, it has been shown that experience, both at the level of the operator and site volumes, is associated with procedural success and lower adverse events, e.g., in-hospital death. Such events are more pronounced in high-risk, complex procedures [18,23,24,25,26]. Hence, the current European Consensus and National Societies guidelines regarding CTO-PCIs established requirements regarding certification for a well-experienced specialist to perform 300 CTO-PCIs in total and maintain more than 50 CTO-PCIs per year [27,28].

In general, despite our findings not underpinning the benefits of second operator involvement, it is worth mentioning that several potential benefits of such an approach were not examined in-depth and are yet to be investigated in the future.

## 5. Conclusions

Due to the retrospective character, the findings of this study have to be considered only as hypothesis-generating. DO CTO-PCI was infrequent and was performed on patients who were more likely to have LMCA lesions or MVD. Operators collaboratively performing CTO-PCIs were more likely to have less experience. Puncture-site bleeding occurred less often in the dual-operator group; however, second-operator involvement had no impact on the technical success of the intervention.

## 6. Study Limitations

This study is limited by several factors. Most importantly, it lacks a randomized design due to its retrospective characteristics. Hence, the results have to be considered solely as hypothesis-generating. Moreover, certain data were not available. This included, for instance, information about the percentage of junior operators involved in DO procedures. Moreover, there was a lack of objective measures concerning CTO complexity, such as the J-CTO index or extensive data regarding the location culprit of the CTO. In addition, the collection of data from multiple centers imposes bias related to the first operators’ divergency. The ultimate recognition of periprocedural complications and ongoing PCI scenarios depends on operator experience, habits, and inclinations. Although our study, on the basis of a large patient cohort, does not support second-operator involvement in CTO cases, more research is necessary to establish an unequivocal consensus on this subject.

## Figures and Tables

**Table 1:** Demographic and clinical characteristics at the baseline in the unmatched population.

Variables	Total (*n* = 27,788)	2 Operators (*n* = 3604)	1 Operator (*n* = 24,184)	*p*-Value
Age, years	66 (60; 73)	66 (60; 73)	66 (60; 73)	0.02
	66.4 ± 9.7	66.1 ± 9.8	66.5 ± 9.7	
Gender, males	19,777 (72.9)	2698 (75.1)	17,079 (72.5)	0.001
Diabetes mellitus	6318 (22.7)	834 (23.1)	5484 (22.7)	0.53
Prior cerebral stroke	769 (2.8)	107 (3.0)	662 (2.7)	0.43
Prior myocardial infarction	11,262 (40.5)	1419 (39.4)	9843 (40.7)	0.13
Prior PCI	13,562 (59.5)	1738 (48.2)	11,824 (48.9)	0.45
Prior CABG	1823 (6.6)	247 (6.9)	1576 (6.5)	0.45
Smoking	3995 (14.4)	598 (16.6)	3397 (14.1)	<0.0001
Psoriasis	91 (0.3)	13 (0.4)	78 (0.3)	0.71
Arterial hypertension	18,259 (65.7)	2575 (71.5)	15,684 (64.9)	<0.0001
Kidney disease	1295 (4.7)	185 (5.1)	1110 (4.6)	0.15
COPD	586 (2.1)	77 (2.1)	509 (2.1)	0.90
Killip class				0.39
I	10,258 (96.5)	1493 (95.9)	8765 (96.6)	
II	326 (3.1)	58 (3.7)	268 (3.0)	
III	30 (0.3)	4 (0.3)	26 (0.3)	
IV	20 (0.2)	2 (0.1)	18 (0.2)	
Killip IV class	20 (0.2)	2 (0.1)	18 (0.2)	0.56

Data are presented as mean ± SD, median (first quartile, third quartile), or counts (percentages). CABG, coronary artery bypass graft; COPD, chronic obstructive pulmonary disease; PCI, percutaneous coronary intervention.

**Table 2:** Demographic and clinical characteristics at the baseline in the propensity score-matched population.

Variables	2 Operators (*n* = 3190)	1 Operator (*n* = 12,760)	*p*-Value
Age, years	65.99 ± 9.75	66.00 ± 9.76	0.96
Gender, males	2406 (75.4)	9608 (75.3)	0.90
Diabetes mellitus	790 (24.8)	3120 (24.5)	0.73
Prior cerebral stroke	96 (3.0)	396 (3.1)	0.83
Prior myocardial infarction	1343 (42.1)	5449 (42.7)	0.55
Prior PCI	1628 (51.0)	6605 (51.8)	0.47
Prior CABG	234 (7.3)	922 (7.2)	0.86
Smoking	562 (17.6)	2172 (17.0)	0.44
Psoriasis			
Arterial hypertension	2304 (72.2)	9155 (71.7)	0.61
Kidney disease	173 (5.4)	681 (5.3)	0.88
COPD	66 (2.1)	269 (2.1)	0.95

Data are presented as mean ± SD, median (first quartile, third quartile), or counts (percentages). CABG, coronary artery bypass grafting; COPD, chronic obstructive pulmonary disease; PCI, percutaneous coronary intervention.

**Table 3:** Procedural indices, outcomes, and operator volumes in the unmatched population.

Variables	Total (*n* = 27,788)	2 Operators (*n* = 3604)	1 Operator (*n* = 24,184)	*p*-Value
Fractional flow reserve	264 (1.0)	37 (1.0)	227 (0.9)	0.61
IVUS	622 (2.2)	92 (2.6)	530 (2.2)	0.17
OCT	81 (0.3)	16 (0.4)	65 (0.3)	0.07
Rotablation	293 (1.1)	29 (0.8)	264 (1.1)	0.12
Mean TIMI grade after PCI	2.16 ± 1.31	2.13 ± 1.33	2.17 ± 1.31	0.18
TIMI grade after PCI				
0	4752 (25.5)	685 (26.6)	4067 (25.3)	0.52
1	418 (2.2)	55 (2.1)	363 (2.3)	
2	473 (2.5)	68 (2.6)	405 (2.5)	
3	12,978 (69.7)	1763 (68.6)	11,215 (69.9)	
TIMI grade 2/3 after PCI	13,451 (72.2)	1831 (71.2)	11,620 (72.4)	0.19
Contrast volume, mL	170 (120; 230)	200 (150; 250)	170 (120; 230)	<0.0001
Radiation dose, Gy	0.92 (0.50; 1.69)	1.05 (0.62; 1.85)	0.90 (0.48; 1.66)	<0.0001
Vascular access				
Femoral	7050 (25.4)	1127 (31.3)	5923 (24.5)	
Radial	20,372 (73.4)	2451 (68.1)	17,921 (74.2)	<0.0001
Other	340 (1.2)	24 (0.7)	316 (1.3)	
Access site crossover	536 (3.1)	86 (3.9)	450 (3.0)	0.02
PCI within bifurcation	1939 (7.0)	242 (6.7)	1697 (7.0)	0.51
Type of stent implanted				
BMS	323 (1.2)	48 (1.3)	275 (1.1)	0.31
BVS	110 (0.4)	24 (0.7)	86 (0.4)	0.006
DES	18,163 (65.4)	2316 (64.3)	15,847 (65.5)	0.14
Drug-eluting balloon	677 (2.7)	105 (3.2)	572 (2.7)	0.08
Implanted stent	18,549 (66.8)	2382 (66.1)	16,167 (66.9)	0.37
Number of implanted stents				
No stent used	9239 (33.3)	1222 (33.9)	8017 (33.2)	
1	14,640 (52.7)	1873 (52.0	12,767 (52.8)	0.73
2	3133 (11.3)	413 (11.5)	2720 (11.3)	
>2	776 (2.8)	96 (2.7)	680 (2.8)	
ASA	7055 (25.4)	927 (25.7)	6128 (25.3)	0.62
UFH	20,563 (74.0)	2589 (71.8)	17,974 (74.3)	0.002
LMWH	784 (2.8)	71 (2.0)	713 (3.0)	<0.001
Bivalirudin	25 (0.1)	5 (0.1)	20 (0.1)	0.30
Thrombolysis during PCI	19 (0.1)	3 (0.1)	16 (0.1)	0.71
Site volume total	6548 (4748.00; 8602.00)	6054 (4232.00; 8745.00)	6747 (4843.00; 8602.00)	0.04
Site volume annual	818.50 (593.50; 1075.25)	756.75 (529.00; 1093.13)	843.38 (605.38; 1075.25)	0.04
Operator volume total	1617 (1096.00; 2261.00)	1368 (857.25; 1970.25)	1676 (1104.00; 2261.00)	<0.0001
Annual operator volume	202.13 (137.00; 282.63)	171.00 (107.16; 246.28)	209.50 (138.00; 282.63)	<0.0001
Operator CTO volume total	92.00 (39.00; 197.00)	80.00 (30.00; 179.00)	93.00 (40.00; 211.00)	<0.0001
Operator CTO volume annual	11.50 (4.88; 24.63)	10.00 (3.75; 22.38)	11.63 (5.00; 26.38)	<0.0001

Data are presented as median (first quartile, third quartile) or counts (percentages), if not stated otherwise. ASA, acetylsalicylic acid; BMS, bare-metal stent; BVS, bioresorbable vascular scaffold; CTO, chronic total occlusion; DES, drug-eluting stent; IVUS, intravascular ultrasound; LMWH, low molecular weight heparin; OCT, optical coherence tomography; PCI, percutaneous coronary intervention; TIMI, thrombolysis in myocardial infarction; UFH, unfractionated heparin.

**Table 4:** Procedural indices, outcomes, and operator volumes in the propensity score-matched population.

Variables	2 Operators (*n* = 3190)	1 Operator (*n* = 12,760)	*p*-Value
Fractional flow reserve	32 (1.0)	120 (0.9)	0.82
IVUS	87 (2.7)	347 (2.7)	1.0
OCT	15 (0.5)	50 (0.4)	0.64
Contrast volume, mL	200 (150, 250)	180 (130, 250)	<0.001
Radiation dose, Gy	1.05 (0.62, 1.84)	0.98 (0.53, 1.76)	<0.001
Vascular access			0.45
Femoral	1066 (33.4)	4122 (32.3)	
Radial	2105 (66.0)	8568 (67.1)	
Other	19 (0.6)	70 (0.5)	
PCI within bifurcation	212 (27.6)	857 (6.7)	0.92
Implanted stent	2134 (66.9)	8522 (66.8)	0.92
ASA	879 (27.6)	3516 (27.6)	1.0
UFH	2304 (72.2)	9441 (74.0)	0.046
LMWH	67 (2.1)	252 (2.0)	0.70
Site volume annual	809.00 (534.75, 1093.12)	818.50 (608.00, 1075.25)	0.75
Operator CTO volume annual	10.00 (4.25, 24.25)	9.88 (4.25, 24.12)	0.75

Data are presented as median (first quartile, third quartile) or counts (percentages), if not stated otherwise. ASA, acetylsalicylic acid; CTO, chronic total occlusion; DES, drug-eluting stent; IVUS, intravascular ultrasound; LMWH, low molecular weight heparin; OCT, optical coherence tomography; PCI, percutaneous coronary intervention; UFH, unfractionated heparin.

**Table 5:** Periprocedural complications in the unmatched population.

Variables	Total (*n* = 27,788)	2 Operators (*n* = 3604)	1 Operator (*n* = 24,184)	*p*-Value
All complications	358 (1.3)	45 (1.3)	313 (1.3)	0.82
Death	22 (0.1)	2 (0.1)	20 (0.1)	0.60
Myocardial infarction	26 (0.1)	5 (0.1)	21 (0.1)	0.34
No-reflow phenomenon	83 (0.3)	9 (0.3)	74 (0.3)	0.56
Cardiac arrest	45 (0.2)	6 (0.2)	39 (0.2)	0.94
Allergic reaction	32 (0.1)	2 (0.1)	30 (0.1)	0.26
Coronary artery perforation	125 (0.5)	19 (0.5)	106 (0.4)	0.46
Puncture-site bleeding	45 (0.2)	2 (0.1)	43 (0.2)	0.09

Data are presented as counts (percentages).

**Table 6:** Periprocedural complications in the propensity score-matched population.

Variables	2 Operators (*n* = 3190)	1 Operator (*n* = 12,760)	*p*-Value
All complications	53 (1.7)	225 (1.8)	0.75
Death	2 (0.1)	12 (0.1)	0.84
Myocardial infarction	5 (0.2)	10 (0.1)	0.33
No-reflow phenomenon	9 (0.3)	45 (0.4)	0.66
Cardiac arrest	9 (0.3)	32 (0.3)	0.91
Allergic reaction	1 (0.0)	15 (0.1)	0.29
Coronary artery perforation	19 (0.6)	67 (0.5)	0.73
Puncture-site bleeding	2 (0.1)	37 (0.3)	0.03

Data are presented as counts (percentages).

## Data Availability

Upon special request.
